# Genome sequence of *Paenibacillus* sp. M.A.Huq-84 isolated from the rhizosphere of pine tree located in Anseong, South Korea

**DOI:** 10.1128/mra.01068-25

**Published:** 2025-11-24

**Authors:** Md. Amdadul Huq, Kwon-Kyoo Kang, M. Mizanur Rahman

**Affiliations:** 1 Department of Life Sciences, College of BioNano Technology, Gachon University 65440, Seongnam-si, Gyeonggi-do, Republic of Korea; 2 Department of Horticultural Life Science, Hankyong National University 105909, Anseong-si, Gyeonggi-do, Republic of Korea; 3 Department of Biotechnology and Genetic Engineering, Faculty of Biological Science, Islamic University 247297, Kushtia, Bangladesh; University of Maryland School of Medicine, Baltimore, Maryland, USA

**Keywords:** genome sequence, *Paenibacillus *sp. M.A.Huq-84, rhizosphere

## Abstract

This study reports the draft genome sequence of *Paenibacillus* sp. M.A.Huq-84, a bacterial strain isolated from the rhizosphere of pine tree located in Anseong, South Korea, in 2021. The genome consists of 8,962,579 base pairs assembled into 99 contigs, encoding 7,675 predicted protein coding genes.

## ANNOUNCEMENT

The genus *Paenibacillus*, which was first described by Ash et al. (1), belongs to the family *Paenibacillaceae*, currently comprises 318 validly published species (2) that have been isolated from a large variety of sources, including foods, water, air, soil, human blood, human feces, milk, and rhizosphere (3–5). During investigations on bacterial biodiversity in the rhizospheric soil of pine tree, a new strain of the genus *Paenibacillus*, designated *Paenibacillus* sp. M.A.Huq-84, was isolated, and its draft genome assembly is presented in this study.

Strain M.A.Huq-84 was isolated from the rhizosphere of a pine tree located in Anseong, South Korea (37° 03′ 16″ N, 127° 30′ 59″ E), during the winter season. Soil samples were collected from the rhizosphere by gently removing soil tightly adhering to the plant roots (collection date: February 10, 2021). One gram of soil was suspended in 9 mL of sterile 0.85% (wt/vol) NaCl solution. The suspension was serially diluted up to a 10^−6^ dilution, and 100 µL of individual dilution was spread onto TSA plates. Then, the plates were placed at 28°C incubator for three days. Single colonies were purified by repeated streaking on fresh TSA plates. The genomic DNA was extracted from the bacterial culture that was grown in TSB for 24 h using Solg Genomic DNA Prep Kit (Solgent, Republic of Korea), according to the manufacturer’s protocol. The DNA fragments were size selected using AMPure XP beads to isolate those within the desired range, typically 200–300 base pairs (bp) for standard Illumina sequencing libraries. The library for sequencing was prepared using the Nextera XT DNA Library Prep Kit (Illumina, San Diego, USA). After library preparation, sequencing was performed on the Illumina HiSeq 3000 platform with paired-end 2 × 150 bp reads, following the standard protocol provided by the manufacturer. Illumina’s bcl2fastq software version 2.20.0 was utilized with default parameters to demultiplex the raw reads and convert them into FASTQ format. Reads were processed using Trimmomatic version 0.38 (6). The genome sequence was assembled by SOAPdenovo v2.04 assembler (7). The genome was annotated using the NCBI Prokaryotic Genome Annotation Pipeline (PGAP) version 6.3 (8–10). The strain was identified using a whole genome-based taxonomic analysis in the Type (Strain) Genome Server (https://tygs.dsmz.de). The genome-to-genome distance (GGDH) bioinformatics tool (11) was used to measure *in silico* DNA-DNA hybridization (isDDH) values. The best match to the strain M.A.Huq-84 genome is the type strain *Paenibacillus foliorum* LMG 31456 (accession GCA_013141765) with *in silico* DNA-DNA hybridization values of 32.2%.

The genome of *Paenibacillus* sp. M.A.Huq-84 is 8,962,579 bp long with a GC content of 45.0%. The assembly contains 99 contigs and 98 scaffolds with a coverage of 43×. The scaffold count is lower by one compared to the number of contigs, due to the merging of two contigs into a single scaffold during the genome assembly process. A total of 7,897 genes were predicted, of which 7,675 are protein-coding genes. The details of genomic features are presented in Table 1.

**TABLE 1:** Genomic features of *Paenibacillus* sp. M.A.Huq-84

Parameter	Result
Description of source	
Location	Anseong, South Korea
Time	2021
Type	Rhizosphere of pine tree
Sequencing summary	
Coverage	43×
Total bases	8,962,579
GC content	45.0%
Assembly report	
Contigs	99
Contig *L*_50_	16
Contig *N*_50_	207.9 kb
Genome length	9 Mb
Annotation report	
Genes (total)	7,897
CDSs (total)	7,782
CDSs (with protein)	7,675
ncRNAs	4
tRNA	89
Quality analysis	
Completeness	99.17%
Contamination	0.67%

## Data Availability

The draft genome sequence of *Paenibacillus* sp. M.A.Huq-84 was deposited in GenBank under accession number JBQWTO000000000. The raw reads are available in SRA under accession number SRR33604982. The version described in this paper is the first version, JBQWTO000000000.1.

## References

[1] Ash C, Priest FG, Collins MD. 1993. Molecular identification of rRNA group 3 bacilli (Ash, Farrow, Wallbanks and Collins) using a PCR probe test. Proposal for the creation of a new genus Paenibacillus. Antonie Van Leeuwenhoek 64:253–260. doi:10.1007/BF00873085 8085788

[2] LPSN. 2025. Available from: https://lpsn.dsmz.de/genus/paenibacillus

[3] Huq MA. 2020. Paenibacillus anseongense sp. nov. a silver nanoparticle producing bacterium isolated from rhizospheric soil. Curr Microbiol 77:2023–2030. doi:10.1007/s00284-020-02086-0 32556479

[4] Akter S, Huq MA. 2018. Biological synthesis of ginsenoside Rd using Paenibacillus horti sp. nov. isolated from vegetable garden. Curr Microbiol 75:1566–1573. doi:10.1007/s00284-018-1561-6 30167766

[5] Huq MA, Kim YJ, Hoang VA, Siddiqi MZ, Yang DC. 2015. Paenibacillus ginsengiterrae sp. nov., a ginsenoside-hydrolyzing bacteria isolated from soil of ginseng field. Arch Microbiol 197:389–396. doi:10.1007/s00203-014-1073-0 25516431

[6] Bolger AM, Lohse M, Usadel B. 2014. Trimmomatic: a flexible trimmer for Illumina sequence data. Bioinformatics 30:2114–2120. doi:10.1093/bioinformatics/btu170 24695404 PMC4103590

[7] Luo R, Liu B, Xie Y, Li Z, Huang W, Yuan J, He G, Chen Y, Pan Q, Liu Y, et al.. 2012. SOAPdenovo2: an empirically improved memory-efficient short-read de novo assembler. Gigascience 1:18. doi:10.1186/2047-217X-1-18 23587118 PMC3626529

[8] Li W, O’Neill KR, Haft DH, DiCuccio M, Chetvernin V, Badretdin A, Coulouris G, Chitsaz F, Derbyshire MK, Durkin AS, Gonzales NR, Gwadz M, Lanczycki CJ, Song JS, Thanki N, Wang J, Yamashita RA, Yang M, Zheng C, Marchler-Bauer A, Thibaud-Nissen F. 2021. RefSeq: expanding the prokaryotic genome annotation pipeline reach with protein family model curation. Nucleic Acids Res 49:D1020–D1028. doi:10.1093/nar/gkaa1105 33270901 PMC7779008

[9] Haft DH, DiCuccio M, Badretdin A, Brover V, Chetvernin V, O’Neill K, Li W, Chitsaz F, Derbyshire MK, Gonzales NR, Gwadz M, Lu F, Marchler GH, Song JS, Thanki N, Yamashita RA, Zheng C, Thibaud-Nissen F, Geer LY, Marchler-Bauer A, Pruitt KD. 2018. RefSeq: an update on prokaryotic genome annotation and curation. Nucleic Acids Res 46:D851–D860. doi:10.1093/nar/gkx1068 29112715 PMC5753331

[10] Tatusova T, DiCuccio M, Badretdin A, Chetvernin V, Nawrocki EP, Zaslavsky L, Lomsadze A, Pruitt KD, Borodovsky M, Ostell J. 2016. NCBI prokaryotic genome annotation pipeline. Nucleic Acids Res 44:6614–6624. doi:10.1093/nar/gkw569 27342282 PMC5001611

[11] Meier-Kolthoff JP, Carbasse JS, Peinado-Olarte RL, Göker M. 2022. TYGS and LPSN: a database tandem for fast and reliable genome-based classification and nomenclature of prokaryotes. Nucleic Acids Res 50:D801–D807. doi:10.1093/nar/gkab902 34634793 PMC8728197

